# Electrochemical aptasensor for lung cancer-related protein detection in crude blood plasma samples

**DOI:** 10.1038/srep34350

**Published:** 2016-10-03

**Authors:** Galina S. Zamay, Tatiana N. Zamay, Vasilii A. Kolovskii, Alexandr V. Shabanov, Yury E. Glazyrin, Dmitry V. Veprintsev, Alexey V. Krat, Sergey S. Zamay, Olga S. Kolovskaya, Ana Gargaun, Alexey E. Sokolov, Andrey A. Modestov, Ivan P. Artyukhov, Nikolay V. Chesnokov, Marina M. Petrova, Maxim V. Berezovski, Anna S. Zamay

**Affiliations:** 1 Krasnoyarsk State Medical University named after prof. V.F. Voino-Yasenecki, Laboratory for Biomolecular and medical technologies, 1 P. Zheleznyaka, Krasnoyarsk 660022, Russia; 2 Institute of Chemistry and Chemical Technology of the Siberian Branch of the Russian Academy of Science, 50/24, Akademgorodok, Krasnoyarsk, 660036, Russia; 3 Design department “Iskra”, 1 Televizornaya, Krasnoyarsk, 660028, Russia; 4 Krasnoyarsk Research Center Siberian branch of Russian Academy of Science 50, Akademgorodok, Krasnoyarsk, 660036, Russia; 5 Krasnoyarsk Regional Clinical Cancer Center named after A.I. Kryzhanovsky 1, Smolenskaya, Krasnoyarsk, 660022, Russia; 6 University of Ottawa, Department of Chemistry, 10 Marie-Curie, D’Iorio Hall, Room 201 Ottawa, ON K1N 6N5, Canada; 7 Institute of Physics named after L.V. Kirenski Siberian Branch of Russian Academy of Science 50/38, Akademgorodok, Krasnoyarsk, 660036, Russia

## Abstract

The development of an aptamer-based electrochemical sensor for lung cancer detection is presented in this work. A highly specific DNA-aptamer, LC-18, selected to postoperative lung cancer tissues was immobilized onto a gold microelectrode and electrochemical measurements were performed in a solution containing the redox marker ferrocyanide/ferricyanide. The aptamer protein targets were harvested from blood plasma of lung cancer patients by using streptavidin paramagnetic beads and square wave voltammetry of the samples was performed at various concentrations. In order to enhance the sensitivity of the aptasensor, silica-coated iron oxide magnetic beads grafted with hydrophobic C8 and C4 alkyl groups were used in a sandwich detection approach. Addition of hydrophobic beads increased the detection limit by 100 times. The detection limit of the LC-18 aptasensor was enhanced by the beads to 0.023 ng/mL. The formation of the aptamer – protein – bead sandwich on the electrode surface was visualized by electron microcopy. As a result, the electrochemical aptasensor was able to detect cancer-related targets in crude blood plasma of lung cancer patients.

Lung cancer is the leading cause of cancer-related death in the world for both men (28%) and women (26%)^1^. The vast majority of lung cancer patients are diagnosed at advanced stages due to a lack of simple disease screening methods for early diagnosis^2^. This establishes the significance of early stage detection of lung cancer.

Biomarkers are molecules released into the blood stream from neoplastic tissues that indicate a change in normal physiological processes^3^. Because of their unique association with genomic changes in cancer cells or other diseases, detection of biomarkers is one of™ the preferred approaches for lung cancer diagnosis^4^. Despite the various available approaches for biomarker detection, new clinically useful methods are needed to overcome the limitations associated with the current methods. Electrochemical-based sensors have been proven as attractive tools for sensitive protein detection^5^ and therefore, hold potential to be successfully applied for tumor biomarker detection in real blood plasma samples.

Square wave voltammetry (SWV) is considered as one of the most advanced voltammetric techniques, which unifies the advantages of pulse techniques, cyclic voltammetry and impedance techniques; it is suitable for analytical application, mechanistic study of electrode processes and electrokinetic measurements^6^. High quality voltammetric data and enhanced sensitivity provided by the specific shape of the potential modulation and the current sampling procedure in SWV, enables effective discrimination against charging currents^7^. Concurrent with a better understanding of electrochemistry and life sciences, sensors and biosensors based on SWV have the potential to serve as next generation point-of-care diagnostic devices^8^.

A number of electrochemical aptamer-based sensors for detection of proteins in blood plasma, serum or whole blood have been previously presented. For example, Lidong Li *et al*. developed a simple and label-free SWV aptasensor for detection of angiogenin (one of the most potent angiogenic factors, related to growth and metastasis of numerous tumors), in which an anti-angiogenin-aptamer was used as a molecular recognition element, and ferro/ferricyanide as a redox probe^9^. Authors compared several methods of angiogenin electrochemical detection such as SWV, electrochemical impedance spectroscopy (EIS) and cyclic voltammetry (CV). As a result, it was found that the SWV method is the most reproducible, sensitive and rapid compared with CV and EIS. The linear range for angiogenin detection was 0.01 nM to 30 nM and the detection limit was 1 pM. An analysis of four plasma samples, diluted 50 times before each trial, from human lung cancer patients was also done using an aptasensor. The authors analyzed these samples further with a human angiogenin immunoassay in order to provide comparative data, which showed that the accuracy of the proposed method was agreeable.

Furthermore, a label-free electronic biosensor for detection of thrombin in blood serum has been previously described^10^. The sensor is constructed by covalently attaching a methylene blue (MB)-labeled, thrombin-binding DNA aptamer to a gold electrode by using well-established self-assembling monolayer chemistry. It was shown that the addition of 64 nM of thrombin to serum caused significant changes in current compared to serum alone.

The development of an RNA-aptamer-based electrochemical sensor with a methylene blue redox label for detection and analysis of tumor necrosis factor-alpha (TNF- α) in whole human blood have been described. The TNF aptamer was modified at the 3′-terminus with a C_6_-disulfide linker and at the 5′-end with an amine group for conjugation with MB redox probe. To stabilize RNA against RNase degradation, phosphorothioates were used and RNA was found to be stable in blood at 37 °C over the course of 10 hours. The detection limit of the aptasensor was 10 ng/mL and the linear range was 100 ng/mL^11^.

Another aptamer-based sensor for the detection of platelet-derived growth factor (PDGF) directly in blood serum has been presented. The sensor was constructed by attaching a MB-modified, PDGF-binding aptamer to a gold electrode via self-assembled monolayer chemistry. In 50% serum (diluted 1:1 with buffer) the signal gain of the sensor was effectively indistinguishable from that obtained in pure buffer despite the presence of 65 mg/ml contaminating serum proteins. In undiluted blood serum the sensor showed a small increase in signal (56%) when the sensor was challenged with 50 nM of platelet-derived growth factor-B subunit homodimer (PDGF-BB)^12^.

A turn-on luminescent aptamer biosensor has been provided for the direct detection of adenosine in undiluted and unprocessed serum, by taking advantage of a terbium chelate complex with long luminescence lifetime to achieve time-resolved detection. The sensor exhibited a detection limit of 60 μM adenosine and demonstrated excellent selectivity that was comparable to that in buffer^13^.

In our present work, LC-18, DNA aptamer clone #18 (CTCCTCTGAC TGTAACCACG TGCCCGAACG CGAGTTGAGT TCCGAGAGCT CCGACTTCTT GCATAGGTAG TCCAGAAGCC) with high affinity and specificity to postoperative lung cancer tissue (K_d_ = 38 nM) and circulating tumor cells^14^ was used to detect tumor associated targets which could serve as biomarkers in blood plasma samples. One of the potential protein targets of LC-18 has been identified as histone H2B^14^ that plays an important role in tumor malignancy and highly ubiquitylated in cancer cells^15^,^16^,^17^. Another potential target has been identified as neutrophil defensin^14^, a protein secreted by neutrophils and higher levels of which are found in several tumor types^18^. In this work, for the first time, crude clinical blood plasma samples from lung cancer patients (LCP) and healthy people (HP) were analyzed by an electrochemical aptasensor using SWV. The data compared the differences of ΔI between LCP and HP blood samples, the means of ΔI for the LCP group was 41.6 μA ± 17.6 μA and for the HP group was 17.0 μA ± 5.6 μA. The maximum and minimum difference between the values of ΔI for LCP and HP blood plasma samples was 49.85 μA and 3.42 μA respectively. The high variability in values between LCP and HP blood plasma samples in some cases and the large deviations for ΔI values can be explained by a fluctuation in protein concentrations for each person^19^.

Moreover, we presented a method for enhancing aptasensor sensitivity for protein detection with IH-SAB 4C–8C (Bioclone Inc.) beads. The hydrophobic beads bind to proteins and promote reduction of the active surface of the electrode resulting in decrease of current. This technique allows detection of protein concentrations 100 times lower compared to protein detection without a sandwich approach with the beads. Aptasensor stability against nuclease activity in blood plasma was also verified. It was shown that LC-18 aptasensor was stable in plasma for over 5 hours.

## Results and Discussion

### Stability of the aptasensor in crude blood plasma

Since most nucleic acids are susceptible to nucleases one of the key problems in analysis of clinical samples is the aptamers’ stability in blood. To verify LC-18 stability in crude blood plasma, aptamer-modified gold electrodes were monitored with and without the presence of blood plasma of lung cancer patient pre-incubated with masking DNA over 5 hours at room temperature.

**Figure 1:**
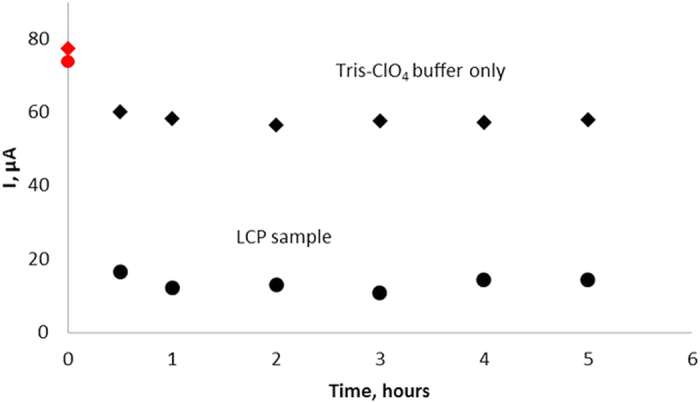
Stability of the LC-18 based aptasensor in Tris-ClO_4_ buffer only (diamonds) and in blood plasma of a lung cancer patient (circles).

Measurements were done as follows. At first we measured electrodes modified with aptamer LC-18 (Fig. 1, point 0 on the axis of timeline) alone. Then, the LCP sample and Tris-ClO_4_ buffer were added on the electrodes and the current at a working electrode was re-measured after 0.5, 1, 2, 3, 4, 5 hours of incubation with the samples (Fig. 1, points 0.5–5 on the timeline axis). As observed from the graph, the current value decreased after incubation with LCP and remained stable for 5 hours in the presence of blood serum. Such good stability may be explained by the addition of an access amount of masking DNA and the fact that the aptamer was selected in the presence of whole blood^14^.

### SWV of purified cancer-related protein using LC-18 based aptasensor

SWV was performed with the following LCPP concentrations: 230, 23, 2.3, 0.23, 0.023, 0.0023 ng/mL. Protein aliquots were pre-incubated on the electrodes for 60 min. After analysis, the electrodes were washed and incubated again with the beads for 60 min.

**Figure 2:**
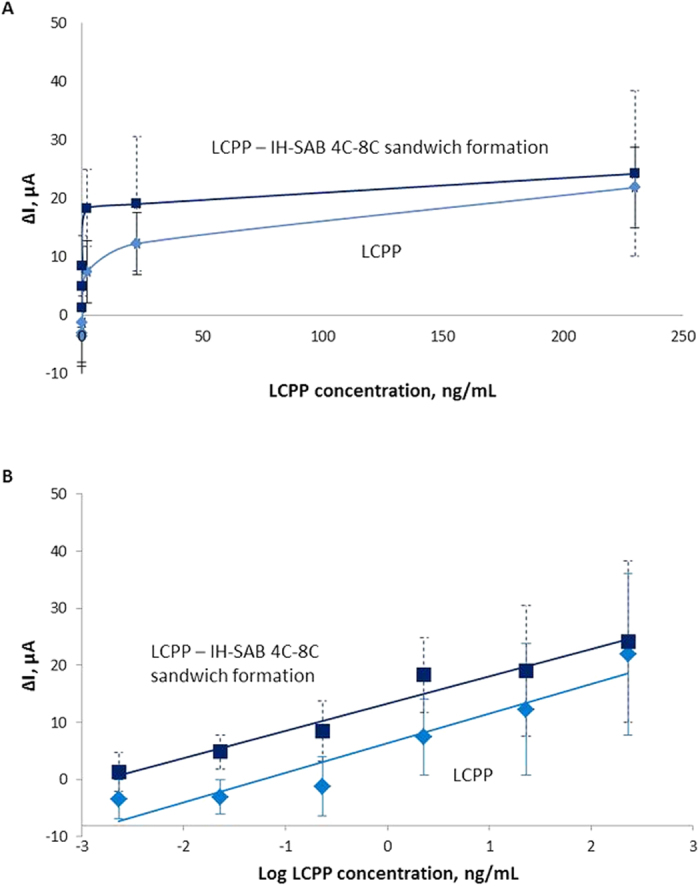
(**A**) Plot of ΔI vs. LCPP (1) and LCPP bound with IH-SAB 4C–8C beads (2) ranging from 230 ng/mL to 0.0023 ng/mL. (**B**) Plot of ΔI vs. LCPP (1) and LCPP bound with IH-SAB 4C–8C beads (2) ranging from 230 ng/mL to 0.0023 ng/mL in logarithmic coordinates.

The plots of ΔI vs. LCPP and LCPP bound with IH-SAB 4C–8C beads ranging from 230 to 0.0023 ng/mL in normal and logarithmic coordinates were depicted in Fig. 2A,B respectively. Where ΔI for electrodes surfaces modified by LC-18-aptamer after adding the LCPP is ΔI_aptamer-target_ = I_max,target_ − I_max,aptamer_. ΔI for electrodes surfaces modified by LC-18-aptamer after adding the LCPP and after adding IH-SAB 4C–8C beads is ΔI_aptamer-IH-SAB 4C–8C_ =I_max, target+IH-SAB 4C–8C_ − I_max, aptamer_.

Calculations were carried out as follows: ΔI_aptamer-target_ was calculated as the difference between the maximal values of current obtained in SWV of electrodes surfaces with tethered DNA-aptamer LC-18 after adding the LCPP in 230, 23, 2.3, 0.23, 0.023, 0.0023 ng/mL and the maximal values of current obtained in SWV of electrodes surfaces with tethered DNA-aptamer LC-18. ΔI_aptamer-IH-SAB 4C–8C_ was calculated as the difference between the maximal values of current obtained in SWV of electrodes surfaces with tethered DNA-aptamer LC-18 after adding the LCPP in 230, 23, 2.3, 0.23, 0.023, 0.0023 ng/mL and after adding IH-SAB 4C–8C beads and the maximal values of current obtained in SWV of electrodes surfaces with tethered DNA-aptamer LC-18.

The detection limit of LCPP obtained with LC-18 based aptasensor was 2.3 ng/mL. Addition of IH-SAB 4C–8C improved the detection limit by 100 times in the linear range between 230 ng/mL to 0.023 ng/mL. The tabulated values of ΔI for LCPP analyzed with the LC-18-based aptasensor are found in Table 1.

IH-SAB 4C–8C magnetic beads are specific to larger molecular weight proteins and have low affinity to intermediate molecular weight proteins bound with aptamers due to the hydrophobic C4 and C8 alkyl groups on the surface. The beads bind to proteins and promote reduction of the active surface of the electrode for admission of redox marker ions resulting in a current decrease.

**Figure 3:**
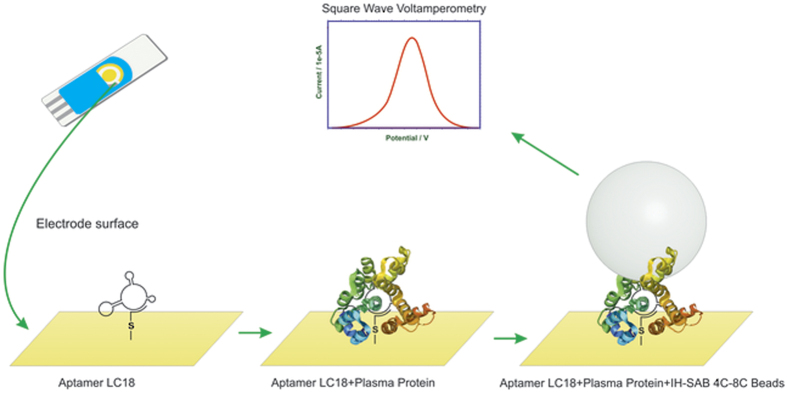
Scheme depicting an aptamer-based sensor for plasma protein detection. (1) 5′-thiolated DNA aptamer LC-18 is self-assembled onto a gold microelectrode. (2) Cancer-related blood plasma protein binds to the immobilized aptamer forming a complex. (3) IH-SAB 4C–8C beads bind to the protein enhancing SWV signal.

In this work, we show that the electrochemical aptasensor was able to detect clinically significant concentrations of cancer-derived proteins. The change in current (ΔI) averages can be used for evaluation of disease biomarker concentration levels. Sandwich formation with the beads enhanced the electrochemical signal for each protein concentration. The additional layer of particles (Fig. 3) bound to the proteins reduced an effective electrode surface and the current decreased. The beads enhanced aptasensor sensitivity by 100 times.

A comparison of the proposed LC-18 based aptasensor with other reported methodologies for detection of proteins in blood plasma, serum or whole blood is presented in Table 2.

### SWV of cancer-related protein in crude blood plasma using LC-18 based aptasensor

It has been previously reported that biosensors can play an important role in early diagnosis of cancer as point-of-care diagnostic devices^18^. The development of crude blood plasma analysis methods is necessary for rapid and easy diagnosis of cancer during screening examinations in clinics as well as in hospitals. Detection of blood plasma oncolytic biomarkers was achieved as follows; blood samples were taken from the patient’s vein in the presence of an anticoagulant, centrifuged, and pre-incubated with masking DNA (1 ng/μL) for 60 min prior to incubation with the surface of the aptasensor for another 60 min. SWV were performed for both LCP blood plasma and HP blood plasma samples, before and after addition of the samples. SWV of LCP and HP blood plasma samples was measured by LC-18-based aptasensors and the data is presented in Figure S2 in the Supporting Information.

**Figure 4:**
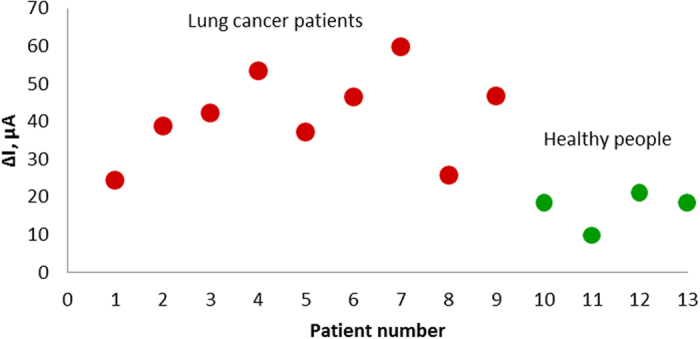
ΔI values in SWV of LCP and HP blood plasma samples measured by LC-18 aptasensor.

Figure 4 shows ΔI values of SWV of LCP and HP blood plasma samples measured by LC-18-based aptasensors. The data shows the differences in ΔI between LCP and HP blood samples analyzed by the aptasensor. The means of ΔI for the LCP group was 41.6 μA and for the HP group was 17.0 μA. The scatters of ΔI in the LCP and HP groups are 54.1% and 53.3% respectively. Wilcoxon Rank Sum Test was used for statistical data analysis on ΔI values in SWV of LCP and HP. P-value for it was 0.005479 which means that the difference between еру average ΔI values in SWV of LCP and HP is statistically significant.

Needless to say, electrochemical signals measured with the plasma samples of each patient would be different due to the high dynamic range in concentrations of cancer-derived biomarkers^20^. It is known that concentrations of the same cancer biomarkers are patient-dependent and may vary for each patient^19^. For example, the level of neuron specific enolase commonly used in lung cancer diagnosis varies from 0 to 170 ng/μL for different patients^19^. Besides this, some proteins known as oncolytic biomarkers may be present in the plasma of healthy persons^3^. Therefore, SWV performed with HP blood plasma samples incubated on the surface of aptasensors also showed variation in current when compared to electrodes with immobilized aptamer only. This was most likely because the same protein targets were present in the HP plasma but at lower concentrations than in the LCP plasma. The tabulated values of ΔI for LCP and HP blood plasma samples analyzed with the LC-18-based aptasensor along with the lung cancer type and stage are found in Table 1. Patients with large cell carcinoma were not included in the analysis because this histological type of lung cancer is quite rare. SCLC and NSCLC are the two main types of lung cancer. Approximately 80% of lung cancer cases are NSCLC which includes: squamous lung cancer (30% cases), adenocarcinoma (30% cases), large cell carcinoma (10% cases) and others (5–10% cases)^21^,^22^.

### Electron microscopy of the aptasensor

**Figure 5:**
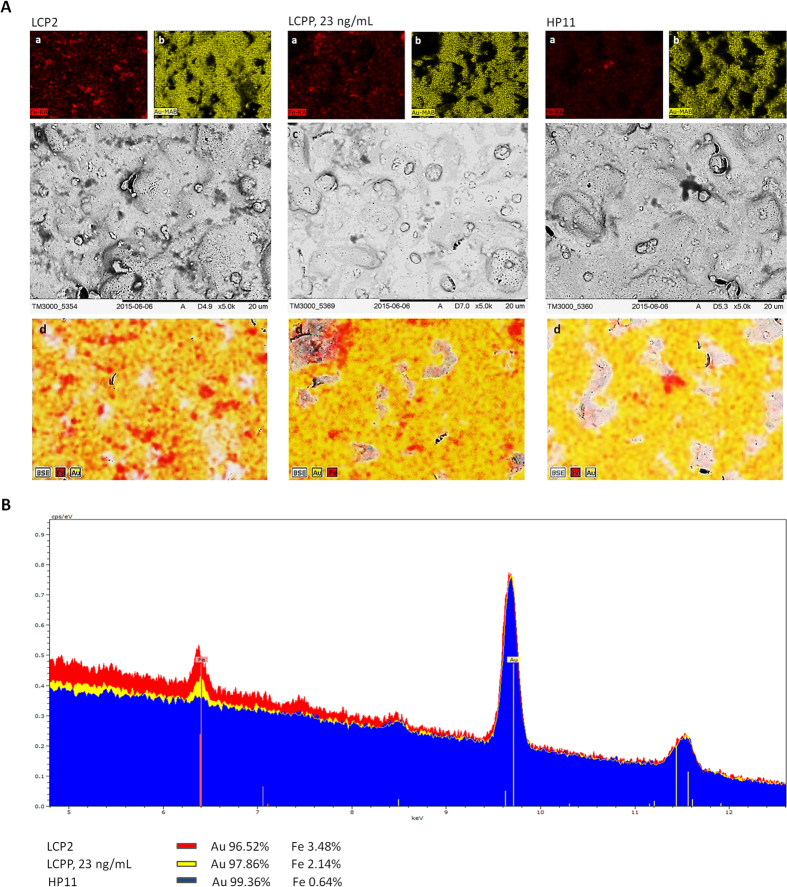
(**A**) Mapping of (a) iron ions and (b) gold ions of the electrodes; (с) contrast EM; (d) overlay of iron and gold ions and contrast EM of the working electrodes surfaces of LC-18-based aptasensors with the formation of targets from the following samples with IH-SAB 4C–8C beads: LCP2 blood plasma sample, LCPP at 23 ng/mL concentration, HP11 blood plasma sample. (**B**) The specters of iron and gold ions from the working electrodes surfaces incubated with LCP2 blood plasma sample, LCPP at 23 ng/mL, HP3 blood plasma sample. The percentage ratios of iron compared to gold are 3.48%, 2.14%, 0.64% for LCP, LCPP and HP respectively.

In order to visualize the presence of cancer-related proteins on the electrode surface, sandwich formation with IH-SAB 4C–8C beads was performed and observed by electron microscopy (EM) (Fig. 5).

The photos obtained by EM showed the highest amount of iron oxide IH-SAB 4C–8C beads on the LC-18-aptamer modified electrode surface after addition of LCP samples. Because the beads bind directly to the proteins, it confirmed that the aptasensor with the LCP samples had the highest concentration of protein targets on its surface as compared with the aptasensor incubated with the HP samples, which had the lowest concentration of protein targets. The fuzzy shape of the beads in the EM images can be explained by the presence of alkyl bonded silica groups on its surface (for binding with proteins), which prevents clear reflection of electrons from oxide molecules, whereby the shape of the beads registered is sufficiently precise.

Besides providing photos of the complex, EM allows to estimate percentage ratios of molecules present in the samples. The percentage ratios of iron compared to gold for LC-18 aptamer modified electrodes after addition of the LCP, LCPP and HP samples were 3.48%, 2.14%, and 0.64% respectively. Thereby, the EM results also show that the LC-18 aptasensor had the highest amount of beads and so the highest concentration of protein target captured by the aptamer on its surface after addition of the LCP sample compared with the aptasensors after addition of the LCPP and HP samples, which complements the SWV data in Table 1.

## Materials and Methods

Methods were carried out in accordance with approved guidelines and the principles expressed in the Declaration of Helsinki. Informed consent was secured from all patients in this study. Using experimental protocol approved by the Local Committee on Ethics of the Krasnoyarsk Regional Clinical Cancer Center named after A.I. Kryzhanovsky and Krasnoyarsk State Medical University, Krasnoyarsk, Russia. Blood for this study were taken from patients who had undergone complete, curative resection of their tumors before the surgery. Samples were transported to the laboratory within 1 hour of collection.

### Aptasensor preparation

Prior to the experiments, the screen-printed gold electrodes (Dropsens, Spain) were washed thoroughly with deionized water then dried with pure N_2_ gas. Thereafter, the 5′-thiolated DNA-aptamer LC-18 (Integrated DNA techonologies, USA) in Dulbecco’s Phosphate-Buffered Saline (DPBS) buffer was immobilized onto a gold electrode surface by incubation for 24 hours at 6 °C in 100% humidity. After backfilling with 0.1 mM 2-mercaptoethanol, crude blood plasma from a lung cancer patient (LCP) or a healthy person (HP) pre-incubated with 1 ng/μL masking DNA (salmon sperm DNA) (Promega Inc., USA) or purified blood plasma proteins harvested from a lung cancer patients (LCPP) were transferred to the electrode surface. Next, a sandwich formation of LCPP with IH-SAB 4C–8C was prepared in order to enhance the electrochemical signal. The scheme describing cancer-related protein detection is depicted in Fig. 3.

### Electrochemical measurements

All measurements were performed at room temperature in an enclosed and grounded Faraday cage. Electrochemical studies were carried out with an electrochemical analyzer (CH Instruments 660D, TX, U.S.). Electrochemical measurements were performed in 20 mM Tris-ClO_4_ buffer (pH 8.6), containing 2.5 mM K_4_Fe(CN)_6_ and 2.5 mM K_3_Fe(CN)_6_. The SWV experiments were performed under the following conditions: the voltage was scanned from −0.2 V to 0.6 V with a potential incremental of 0.005 V, an amplitude of 0.025 V, a frequency of 15 Hz and a quiet time of 2 s.

### Harvesting of LC-18 targets from blood plasma

In order to measure the concentration of cancer-related biomarkers specific to aptamer LC-18, protein targets were harvested from blood plasma obtained from LC patients (Figure S1). More specifically, the plasma samples were pre-incubated with masking DNA (1 ng/μL) to prevent non-specific binding prior to incubation with 5′-biotinylated aptamer conjugated with streptavidin coated MagneSphere Paramagnetic Particles (Promega Inc., USA). Thereafter, protein targets that bound with the MagneSphere particles were pulled onto the wall of the tube, the liquid was discarded as it contained among other things, non-bound proteins, and then a solution of urea was added to the MagneSphere to dissociate the proteins from the aptamers. Finally, collected blood plasma proteins from LC patients were washed with DPBS and concentrated with 30 kDa cut-off filters, and the concentration was measured with a NanoDrop spectrophotometer.

### Electron microscopy

An electron microscopy (Hitachi TM3000, Japan) was used to visualize the sandwich formation of cancer-related proteins bound with LC-18 aptamer on the electrodes surfaces with IH-SAB 4C–8C beads. In order to estimate percentage ratios of molecules present on the electrodes EM spectra were processed with the software Quantax 70 (Bruker) for Hitachi TM3000.

## Conclusion

The development of biosensors for detection of serum biomarkers provides an inexpensive, non-invasive and easy tool for clinical use and routine diagnostics^23^. Known cancer-related biomarkers still lack sufficient specificity and sensitivity for use in early cancer diagnosis^24^. This work clearly illustrates cancer-related protein detection in crude blood plasma samples of lung cancer patients. Aptamer LC-18 with high affinity to lung cancer tissue and circulating tumor cells in blood was used to construct an aptasensor to detect cancer-related proteins in blood plasma samples.

## Additional Information

**How to cite this article**: Zamay, G. S. *et al*. Electrochemical aptasensor for lung cancer-related protein detection in crude blood plasma samples. *Sci. Rep.* **6**, 34350; doi: 10.1038/srep34350 (2016).

## Supplementary Material

Supplementary Information

## Figures and Tables

**Table 1:** ΔI values in SWV for LCP and HP blood plasma samples and LCPP analyzed by LC-18 aptasensor.

Sample group	Sample name	ΔI_aptamer-target_, μA	ΔI_aptamer-IH-SAB 4C–8C,_ μA	Lung cancer type	Lung cancer stage	MTS	Other diseases	WBS^*^ 10^9^/L	NEU^*^ 10^9^/L	HGB^*^ g/L	Age	Sex
Lung cancer patients’ blood plasma	1	24.52	—	Small cell	IV	Brain	Duodenal ulcer, chronic bronchitis	9.08	5.38	131	59	M
	2	38.73	—	Squamous	IIA	—	Chronic obstructive pulmonary disease	8.5	5.82	119	67	F
	3	42.27	—	Adenocarcinoma	IIIB	—	Chronic obstructive pulmonary disease, duodenal ulcer	8.68	4.70	106	65	M
	4	53.46	61.17	Adenocarcinoma	IIIA	—	Hypertonic disease	7.04	5.21	125	52	M
	5	37.23	38.83	Small cell	IIA	—	Cardiac ischemia, myocardial infarction stomach ulcer	6.32	6.11	106	69	M
	6	46.40	61.91	Squamous	IIIB	—	Chronic obstructive pulmonary disease	17.74	4.49	139	66	M
	7	59.70	61.17	Squamous	IIIB	—	Transient ischemic attack	12.11	5.45	112	45	M
	8	25.67	28.89	Adenocarcinoma	IB	—	Paroxysmal atrial fibrillation, gastrectomy for peptic ulcer disease	10.19	6.79	146	67	M
	9	46.84	52.21	Squamous and adenocarcinoma (mixed type)	IA	—	Hypertonic disease, duodenal ulcer, chronic bronchitis	7.87	5.02	116	76	M
Healthy persons’ blood plasma	10	18.37	—	—	—	—	—	6.61	4.25	142	62	M
	11	9.85	—	—	—	—	—	4.01	2.95	160	30	M
	12	21.10	—	—	—	—	—	7.08	5.31	125	36	F
	13	18.51	—	—	—	—	—	5.15	2.37	135	60	F
Concentration of purified proteins (ng/mL) harvested from blood plasma obtained from patient LC6	230	21.90	24.23	Squamous	IIIB	—	Chronic obstructive pulmonary disease	17.74	4.49	139	66	M
	23	12.31	19.05									
	2,3	7.48	18.32									
	0,23	−1.15	8.46									
	0,023	−3.03	4.88									
	0,0023	−3.44	1.3									
Concentration of purified proteins (ng/mL) harvested from blood plasma obtained from patient LC8	230	17,4	11,01	Adenocarcinoma	IB	—	Paroxysmal atrial fibrillation, gastrectomy for peptic ulcer disease	10.19	6.79	146	67	M
	23	13,36	12,96									
	2,3	1,74	11,34									
	0,23	−5,25	2,68									
	0,023	−4,57	1,67									
	0,0023	−7,76	−2,4									
Concentration of purified proteins (ng/mL) harvested from blood plasma obtained from patient LC9	230	18,4	22,57	Squamous and adenocarcinoma (mixed type)	IA	—	Hypertonic disease, duodenal ulcer, chronic bronchitis	7.87	5.02	116	76	M
	23	6,55	11,89									
	2,3	8,25	19,15									
	0,23	9,19	9,8									
	0,023	2,53	7,6									

*WBS – level of white blood cells in blood, NEU – level of neutrophils in blood, HGB – level of hemoglobin in blood.

**Table 2:** Comparison of proposed biosensor with other reported methodologies for detection of proteins in blood plasma, serum or whole blood.

Sensing target	Detection limit	Biosensor type	Reference
Angiogenin	1 pM	SWV based aptamer biosensor	9
Thrombin	6.4 nM	MB based aptamer biosensor	
Tumor necrosis factor-alpha	10 ng/mL	MB based aptamer biosensor	11
Platelet-derived growth factor	1.25 ng/mL (50 pM)	MB based aptamer biosensor	12
Adenosine	60 μM	Turn-on luminescent aptamer biosensor	13
Histone H2B Neutrophil defensin	2.3 ng/mL (without IH-SAB 4C–8C) 0.023 ng/mL (with IH-SAB 4C–8C)	SWV based aptamer biosensor	Our work
