# Supplementary material for: Electrochemical aptasensor for lung cancer-related protein detection in crude blood plasma samples
Source: Sci Rep. 2016 Oct 3;6:34350. doi: 10.1038/srep34350 (PMC5046130; doi:10.1038/srep34350)
Supplement: Supplementary Information [file srep34350-s1.doc]

**Supplementary Information**

**Electrochemical aptasensor for lung cancer-related protein detection in crude blood plasma samples**

Galina S. Zamay^1,2^, Tatiana N. Zamay^1^, Vasilii A. Kolovsky^7^, Alexandr V. Shabanov^3^, Yury E. Glazyrin^1,2^, Dmitry V. Veprintsev^1^, Alexey V. Krat^4^, Sergey S. Zamay^3^, Olga S. Kolovskaya^1^, Ana Gargaun^6^, Alexey E. Sokolov^5^, Andrey A. Modestov^4^, Ivan P. Artyukhov^1^, Nikolay V. Chesnokov^2^, Marina M. Petrova^1^, Maxim V. Berezovski^6^, Anna S. Zamay*^1,2^

^1^Krasnoyarsk State Medical University named after prof. V.F. Voino-Yasenecki, Laboratory for Biomolecular and medical technologies, 1 P.Zheleznyaka, Krasnoyarsk, Russia 660022

^2^Institute of Chemistry and Chemical Technology of the Siberian Branch of the Russian Academy of Science, 50/24, Akademgorodok, Krasnoyarsk, Russia, 660036

^3^Krasnoyarsk Research Center Siberian branch of Russian Academy of Science
50, Akademgorodok, Krasnoyarsk, Russia, 660036

^4^Krasnoyarsk Regional Clinical Cancer Center named after A.I. Kryzhanovsky
1, Smolenskaya, Krasnoyarsk, Russia, 660022

^5^Institute of Physics named after L.V. Kirenski Siberian Branch of Russian Academy of Science
50/38, Akademgorodok, Krasnoyarsk, Russia, 660036

^6^University of Ottawa, Department of Chemistry, 10 Marie-Curie, D'Iorio Hall, Room 201
Ottawa, ON, Canada K1N 6N5

^7^ Design department “Iskra”, 1 Televizornaya, Krasnoyarsk, Russia, 660028

*Correspondent author: Anna S. Zamay, E-mail: aszamay@gmail.com, annazamay@yandex.ru, Tel. +7 903 923 84 02

Figure S1.


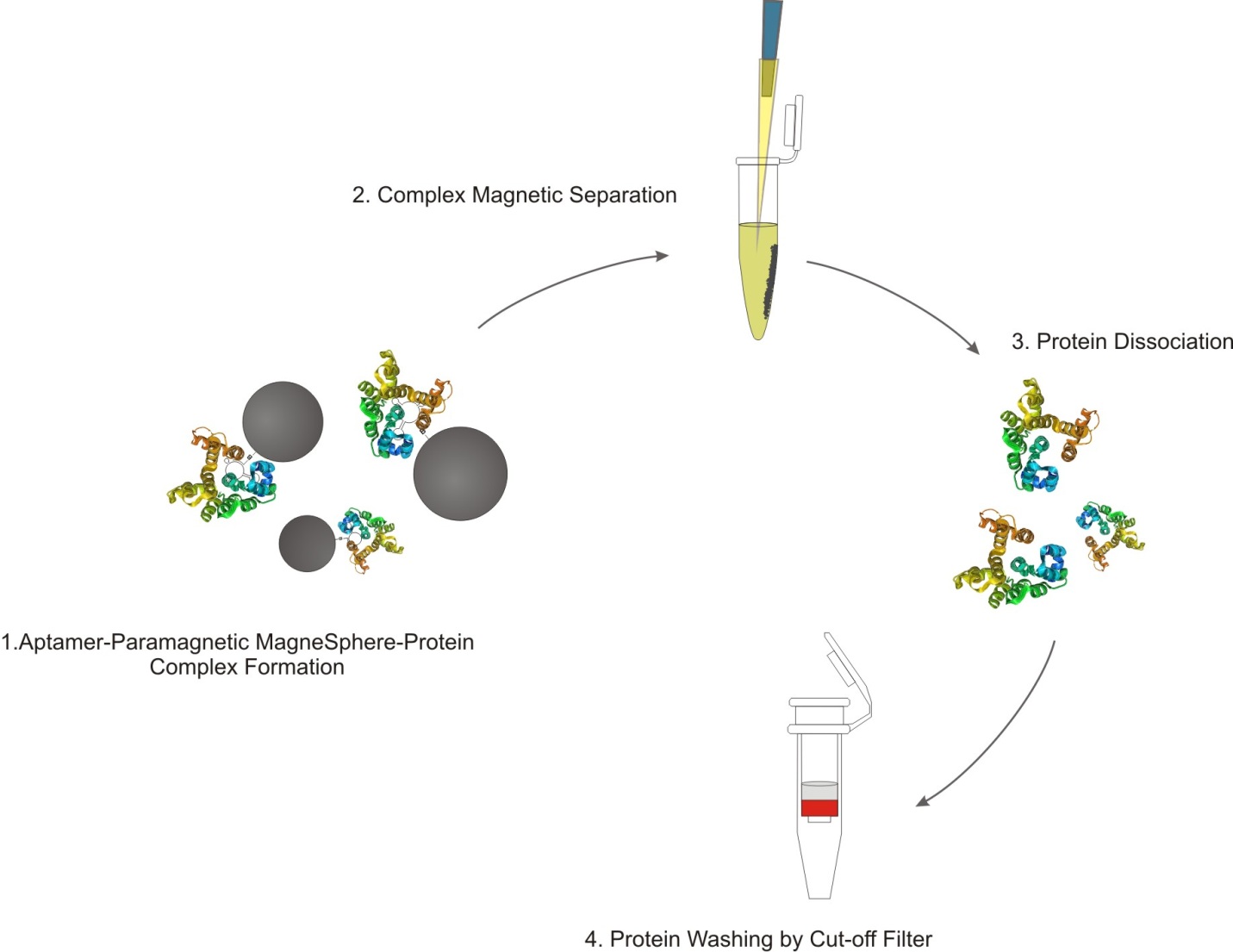


Figure S1. Scheme describing cancer-related proteins harvested from a blood plasma sample. (1) Blood plasma sample was incubated for 30 min with masking DNA (1 ng/µl), followed by incubation with 50 nM 5’-biotinylated aptamer conjugated with Streptavidin MagneSphere Paramagnetic Particles for 60 min at room temperature. (2) Proteins bound with MagneSpheres were pulled onto the wall of the tube. (3) Proteins were dissociated from aptamer-coated beads by incubation with 30 µL of 8 M urea solution for 60 min at room temperature. (4) LCP proteins were washed with DPBS and concentrated by a 30 kDa cut-off filter.

Figure S2.


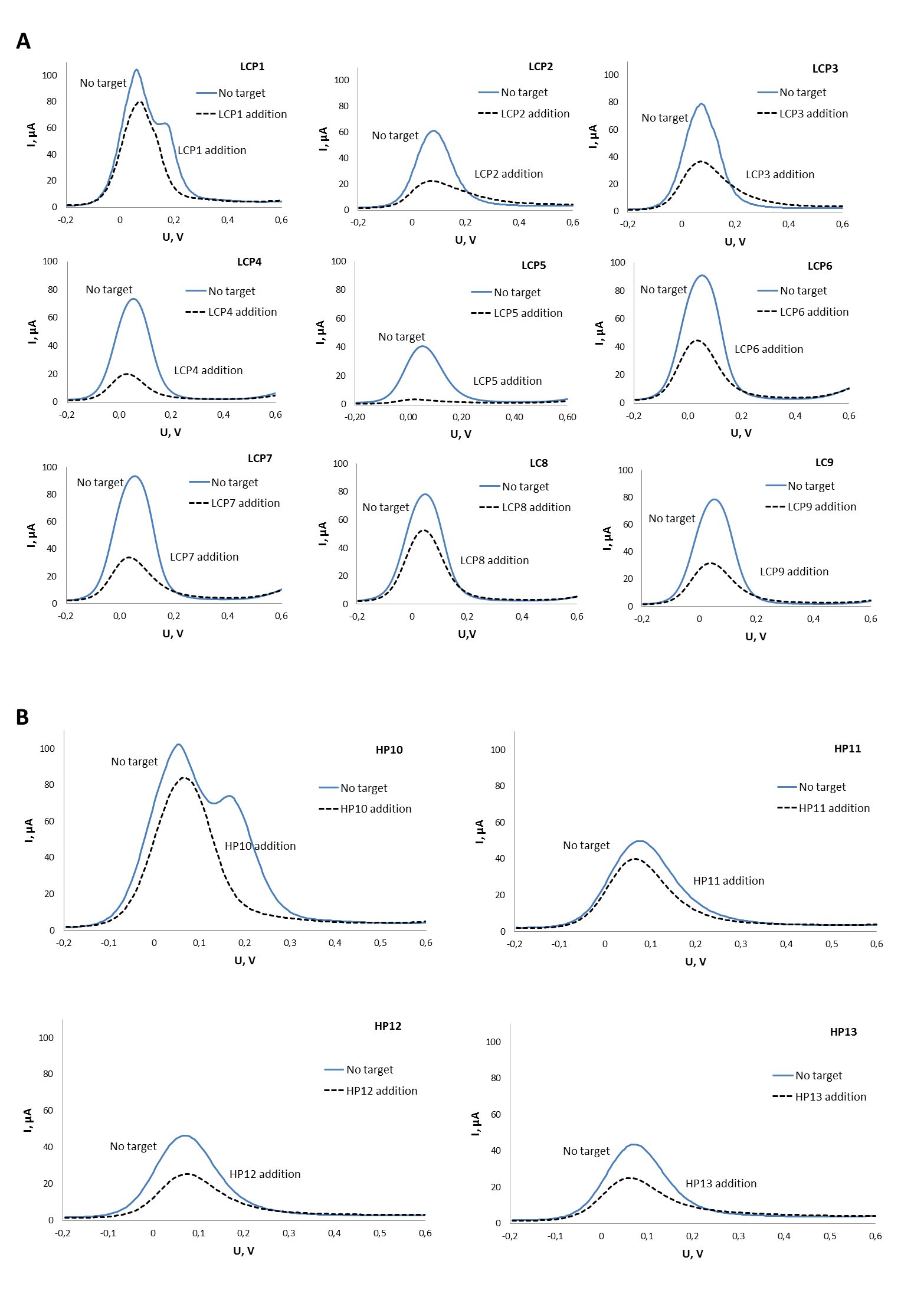


Figure S2. (A) SWV carried out with LCP blood plasma samples and measured with LC-18-based aptasensor. (B) SWV of HP blood plasma samples and measured with LC-18-based aptasensor.
